# Characterization of the complete chloroplast genome sequence of *Stipa bungeana* (Poaceae), an important forage grass in the temperate steppe of Northern China

**DOI:** 10.1080/23802359.2022.2139161

**Published:** 2022-11-11

**Authors:** Zhanjun Wang, Ying Tian, Bo Ji, Wangsuo Liu

**Affiliations:** a Institute of Forestry and Grassland Ecology, Ningxia Academy of Agriculture and Forestry Science, Yinchuan, China; b Ningxia Key Laboratory of Desertification Control and Soil and Water Conservation, Ningxia Academy of Agriculture and Forestry Science, Yinchuan, China; c School of Agriculture, Ningxia University, Yinchuan, China; d Ningxia Technical College of Wine and Desertification Prevention, Yinchuan, China

**Keywords:** Complete chloroplast genome, *Stipa bungeana*, phylogenetic analysis

## Abstract

*Stipa bungeana* Trin. 1833 is an important forage grass in Poaceae, widely distributed in the temperate steppe of Northern China, with strong grazing tolerance and feeding value. In this study, we performed the complete chloroplast (cp) genome sequence of *S. bungeana* to explore its phylogenetic position with other *Stipa*. The results showed that the circular complete cp genome of *S. bungeana* was 137,759 bp in length, including a large single copy (LSC) of 81,652 bp, a small single copy (SSC) of 12,817 bp, and two inverted repeats (IR) of 21,645 bp. The GC content accounts for 43.71% and annotated 134 single genes, which include 87 protein-coding genes, eight rRNA genes, and 39 tRNA genes. Maximum-likelihood (ML) phylogenetic tree suggested that the *S. bungeana* was closely related to other *Stipa* except for *S. purpurea*.

*Stipa bungeana* Trin. 1833, is an important forage grass of *Stipa* in the family Poaceae, widely distributed in the temperate steppe of northern China, and it also is a typical representative species of the Eurasian steppe (Kuo 1987). The main difference between *S. bungeana* and other *Stipa* is that the awn needle of *S. bungeana* is 3–5 cm long, slightly curved, and shiny as hair, 2-geniculate, column 1–1.5 cm to first bend, 0.5–1.0 cm to second bend (Kuo and Sun 1982; Figure 1). *S. bungeana* is a typical bottom grass, with the characteristics of trampling resistance, suitable for use in grazing land, this species germinates earlier in spring and has stronger tillering ability, it is rich in nitrogen-free extract and crude fiber, the cattle and sheep are more fond of feeding and tend to gain weight (Kuo 1987; Jia 1997). To assess the relationship between genetic diversity and geographical distribution, previous studies have reported that the genetic diversity of *S. bungeana* is higher at the species level than at the population level, and which is no significant relationship between the genetic distance and geographical distance (Jing et al. 2013). The wild *Stipa* species in the natural ecosystem are prone to hybridization, the studies have found that the offspring of this natural hybridization had a great variation in morphology, and the taxonomic status of *S. bungeana* is not certain from the genome, it should belong to the section *Leiostipa*, but from the perspective of genetics, it is closer to *S. breviflora* of the section *Barbatae* (Baiakhmetov et al. 2020). A study of the hybrid-complex for *S. heptapotamica* showed that the hybridization between the wild *Stipa* each other of Poaceae is an introgression event (Nobis et al. 2019). However, the uncertainty of the taxonomic status of *S. bungeana* (Baiakhmetov et al. 2020) and the evidence for the chloroplast (cp) whole genome and its phylogenetic status still need to explore. Angiosperm Phylogeny Group (APG) classification system facilitates our understanding of plant evolution and classification (Chase et al. 2016), such as gene markers. The gene fragments (*accD*, *matk*, *rps16-trnQ*, *psbA-trnH*, etc) in plastids are commonly used to mark phylogenetic status and taxonomic attributes of species (Mishra et al. 2016; Van Do et al. 2021). It has been reported that these barcodes can be used as a species identification tool for *Stipa* of Poaceae, such as *S. pennata* (Krawczyk et al. 2018), *S. capillata* (Baiakhmetov et al. 2021), and *S. lipskyi* (Myszczyński et al. 2016). These highlight the importance of cp genomes in taxonomy. Thus, we determined the complete cp genome of *S. bungeana* to analyze and confirm a phylogenic position (Nobis et al. 2019), as well as to provide useful information for further studies.

**Figure 1.**
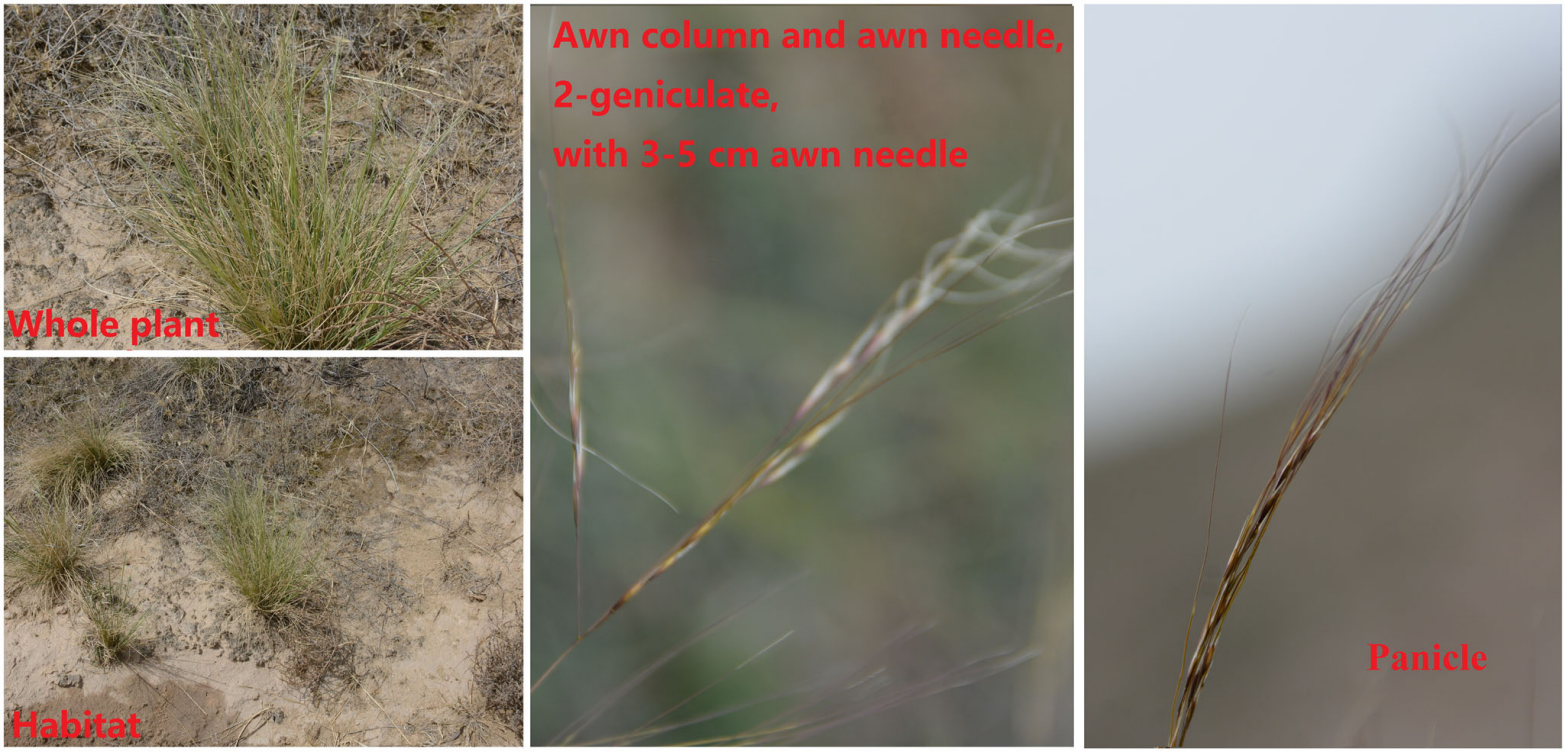
Morphological characteristics of *S. bungeana.*

Fresh leaves of *S. bungeana* were sampled from desert steppe in eastern and western Ningxia, China (38°7′38.57 E, 106°34′27.31 N, alt. 1152 m), and samples are stored in dry ice buckets. The voucher specimen (2022-Stipa_bungeana001) was deposited at the herbarium of the Institute of Forestry and Grassland Ecology, Ningxia Academy of Agriculture and Forestry Science (http://www.nxaas.com.cn/, Wangsuo Liu, email: liuwangsuo@sina.com). Total genomic DNA was extracted according to the modified CTAB method of Stefanova et al. (2013), and stored in the −80 °C refrigerator in the laboratory. The genome sequencing was conducted by Illumina Hiseq 2500 at Biomarker Technologies Corporation. 25,873,239 paired-end reads were obtained and 20,893,700 reads were used to assemble into reference sequences ranging in length from 50 to 151 bp after trimming. The high-quality sequences were assembled by the assembler SPAdes3.11.0 (Nurk et al. 2013). The annotation was performed by Plann (Huang and Cronk 2015). Then, the complete cp genome map was drawn by OGDRAW-Draw Organelle Genome Maps (https://chlorobox.mpimp-golm.mpg.de/OGDraw.html) (Greiner et al. 2019). The sequence of *S. bungeana* complete cp genome has been submitted to the NCBI database (accession number ON854660).

The *S. bungeana* cp genome is 137,759 bp in length, including two inverted repeat regions (21,645 bp) that are separated by a small single-copy region (12,817 bp) and a large single-copy region (81,652 bp). The cp genome contains 134 single genes, including 87 protein-coding genes (CDS), eight rRNA, and 39 tRNA genes (Figure 2). The GC content is 43.71%. Most of these genes are single copy, whereas eight CDS (*rps19*, *rps15*, *rps7*, *rp123*, *rp12*, *ndhH*, *ycf68*, *ndhB*), 10 tRNAs (*trnA-UGC*, *trnH-GUG*, *trnI-CAU*, *trnI-GAU*, *trnL-CAA*, *trnM-CAU*, *trnN-GUU*, *trnR-ACG*, *trnV-GAC*, *trnfM-CAU*) and four rRNAs (*rrn-16S*, *rrn-23S*, *rrn4.5*, *rrn5*) occur as double copies.

**Figure 2.**
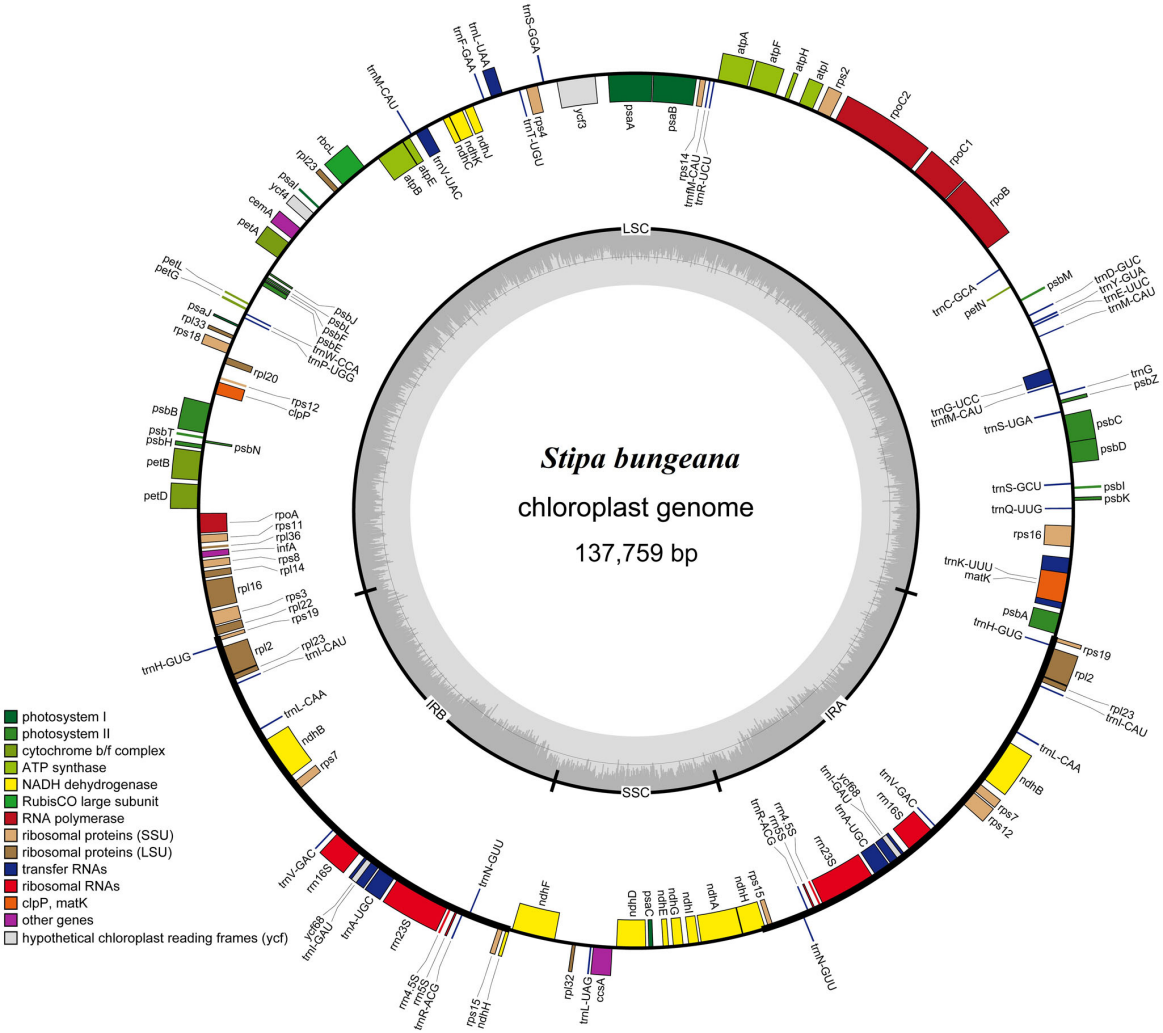
Gene map of *S. bungeana* chloroplast genome. Genes shown inside the circle indicate that the direction of transcription is clockwise, while those shown outside are counterclockwise. Different groups of functional genes are indicated in different colors. The GC content is shown in the dashed area in the inner circle.

The cp genomes of 21 *Stipa* in Poaceae and two outgroups were downloaded from the NCBI database and aligned with *S. bungeana* using MAFFT-7.037 (Katoh and Standley 2013). A maximum-likelihood tree was constructed by MEGA-X (Kumar et al. 2018) based on 24 species (Figure 3). The phylogenetic tree showed that the *S. bungeana* clustered into a clade with most species of *Stipa* except *S. purpurea*. This change may be related to the higher genetic differentiation value between *S. bungeana* and other *Stipa* species, or the event of genetic drift (Jing et al. 2013). Krawczyk et al. (2018) analyzed 21 newly sequenced complete plastid genomes from 19 groups of *Stipa* through the gene fragment analysis, and they found that the multilocus barcodes composed of *ndhH*, *rpl23*, *ndhF-rpl32*, *rpl32-ccsA*, *psbK-psbI* and *petA-psbJ* for *Stipa* were performed the best, but the effectiveness was less than 70% of the analyzed taxa, indicating that these markers did not apply to all *Stipa*. Hybridization among species of *Stipa* in different habitats may have resulted in different highly variable gene segments (Baiakhmetov et al. 2020). In the view of the phylogenetic clustering tree, although it was related to other *Stipa*, it clustered in a single clade (Figure 3). This clustering may confirm that the existence of introgression in *Stipa*, which is likely to be closely related to hybridization (Nobis et al. 2019). In our study, *S. bungeana* was distributed in water-deficient dunes, therefore, environmental filtration and interspecific hybridization were inevitable, and perhaps these factors are interesting areas of future research to explore genetic changes in the same species of *Stipa*. Our study provides ideas for future studies of *S. bungeana* from the perspective of the cp genome.

**Figure 3.**
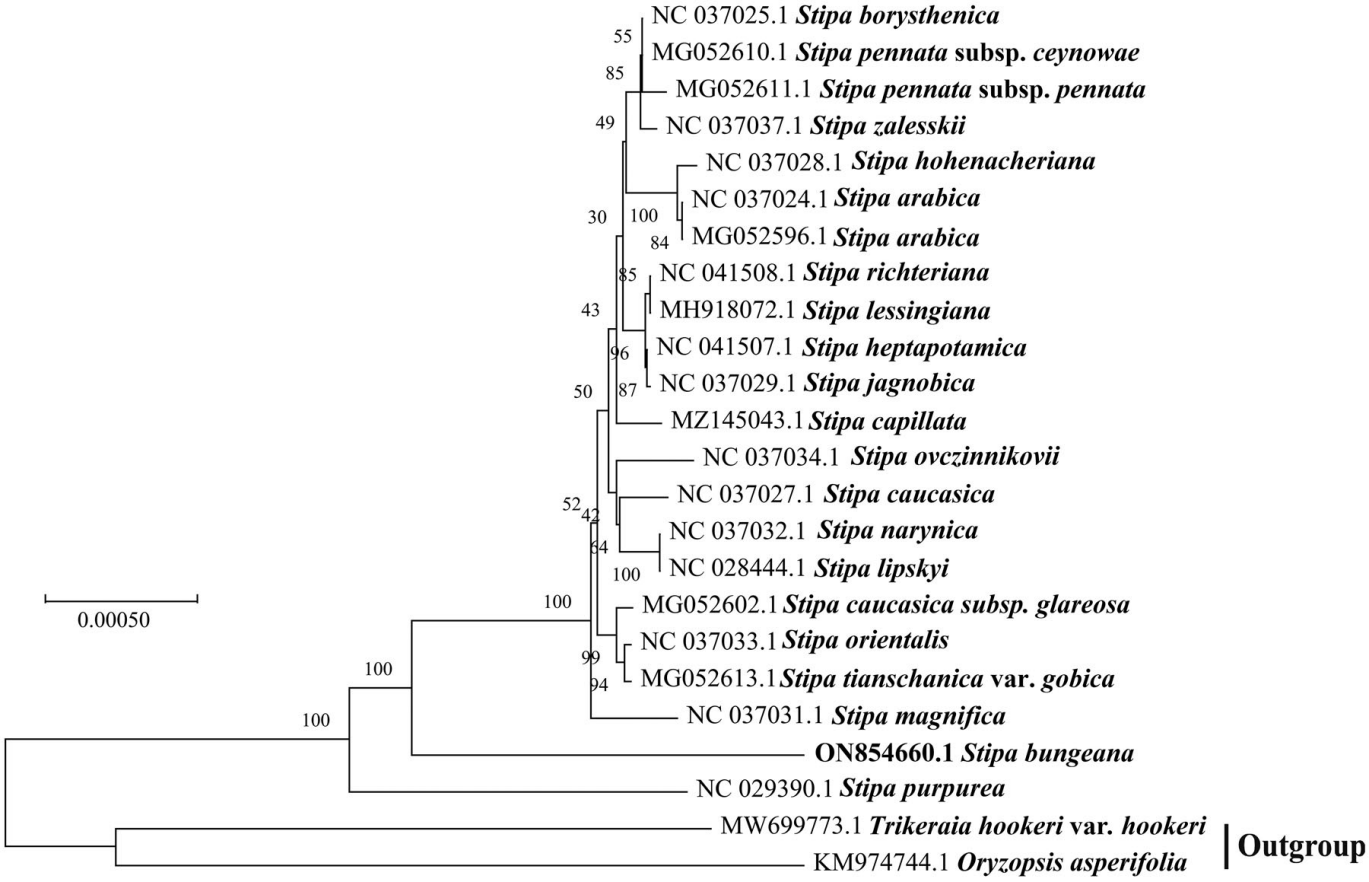
Maximum-likelihood tree based on 24 complete cp genomes. The number on each node represents bootstrap values.

## Data Availability

The data that support the findings of this study are openly available in the NCBI database at https://www.ncbi.nlm.nih.gov/, reference number ON854660. The associated BioProject, SRA, and Biosample numbers are PRJNA852531, SRR19959630, and SAMN29333018, respectively.
